# Epidemiologic-economic models and the One Health paradigm: echinococcosis and leishmaniasis, case studies in Veneto region, Northeastern Italy

**DOI:** 10.1016/j.onehlt.2019.100115

**Published:** 2019-11-27

**Authors:** Massimo Canali, Maurizio Aragrande, Andrea Angheben, Gioia Capelli, Michele Drigo, Federico Gobbi, Francesca Tamarozzi, Rudi Cassini

**Affiliations:** a Department of Agricultural and Food Sciences, University of Bologna, Bologna, Italy; b Infectious and Tropical Diseases, IRCCS Hospital Sacro Cuore – Don Calabria, Negrar, Verona, Italy; c National reference centre/OIE collaborating centre for diseases at the animal-human interface, Istituto Zooprofilattico Sperimentale delle Venezie, Legnaro (PD), Italy; d Department of Animal Medicine, Production and Health, University of Padova, Padova, Italy; e WHO Collaborating Centre for Epidemiology, Detection and Control of Cystic and Alveolar Echinococcosis, Department of Infectious Diseases, Istituto Superiore di Sanità, Rome, Italy

**Keywords:** Epidemiologic-economic models, Cystic echinococcosis, Leishmaniasis, Neglected zoonosis, Epidemiology of parasitic zoonoses, Health economics, Animal health economics, One health economics

## Abstract

Epidemiology and health economics have systemic interdependencies. The identification of the economic outcomes of any disease is operated by overlapping its epidemiology with the economic functions of the impacted entities. This communication presents two epidemiologic-economic models designed to evaluate the economic burden of cystic echinococcosis and leishmaniasis in Veneto (Northeastern Italy). Following a One Health approach, the research integrates expertise from different disciplines and institutions and fulfilled its first stage by defining the relevant cost categories and the data collection strategy for the two diseases in the study area. The two models identify the relevant epidemiological factors and the economic outcomes of infections in both animals and humans. The results, visualized in flow charts indicating the types of costs associated with these zoonoses, will guide data collection and the epidemiologic and economic assessment in the next research stages. This experience shows that One Health methods, although still innovative or unusual in many scientific and professional contexts, can be applied by using relatively limited resources and already available professional skills.

## Highlights

• An economic evaluation of c. echinococcosis and leishmaniasis in a hypo-endemic area was approached with systems-thinking.
• The research team and an external network of experts integrated the necessary interdisciplinary knowledge.
• Two epidemiologic-economic models were designed to identify relevant cost categories and data sources.
• This experience shows that One Health methods can be applied with limited resources and skills already available.

## 1 Introduction: seeking a One Health approach for echinococcosis and leishmaniasis in a hypo-endemic area

This communication concerns the methodology applied to implement a research project on cystic echinococcosis (CE) and leishmaniasis in Veneto, a region of 4.9 million inhabitants in Northeastern Italy. CE is originated by infections with the larval stage of the tapeworm *Echinococcus granulosus* sensu *lato*. Canids (definitive hosts) harbour adult parasites, while larvae develop in several livestock species (intermediate hosts) and also in humans (accidental intermediate hosts) [[1], [2], [3], [4]]. The only agent of human leishmaniasis (HumL) in Italy is *Leishmania infantum*, a protozoan transmitted by *Phlebotomus* sandflies that can determine important illness, especially in non-fully immune-competent individuals. In the study area, as in all Southern Europe, dogs represent the main reservoir of this pathogen, which also causes canine leishmaniasis (CanL) [[5], [6], [7], [8]]. Both CE and leishmaniasis have high public health impacts worldwide [3,5], recognise domestic animals as reservoirs, and are considered emerging and hypo-endemic in Veneto, with verified circulation in animal populations [4,8,9] and human cases recorded [10,11].

The main objectives of the research – funded through the person-time costs of the experts from the different institutions involved and a small university grant – are (i) the evaluation of the costs currently bore by the population and the health services of the region for the two diseases, and (ii) the assessment of possible interventions. The research encompasses three stages: the first includes an analysis of the epidemiological characteristics of the two diseases in the region, the identification of their costs, and the design of a data collection strategy; the second consists in data collection and epidemiologic and economic assessments; and the last covers result analysis and evaluation of possible measures to inform public health decision-makers. The research approach is inspired by One Health concepts and practices [12,13], in particular, systems thinking and inter/trans-disciplinarity, which shaped both the selection of the heterogeneous expertise involved in the study and the work organization. Until now, the first stage of the project has been completed, by identifying, respectively for CE and leishmaniasis, the interrelations of the relevant disease impacts on animal and human health, the environment, and the socioeconomic domain. Here we present the methodological aspects of the project and two epidemiologic-economic models (EEMs) that describe, for each disease, the interdependencies of the outcomes and their economic consequences, up to the definition of data collection strategies for cost evaluation.

## 2 Integrated expertise and systemic interdependencies between epidemiology and health economics: the construction of epidemiologic-economic models

Inter/trans-disciplinarity and systems thinking are functionally interrelated and increasingly applied to health issues [[14], [15], [16], [17]] especially in the One Health context [12,15,[18], [19], [20], [21], [22], [23]]. In this study, they allowed to size the epidemiologic and socioeconomic complexity of CE and leishmaniasis through the integration of interdisciplinary expertise and the elaboration of two EEMs.

Seven specialists in human and veterinary parasitology, and economics, from different academic and health research institutions and hospitals form the research team (RT) covering the basic set of disciplines needed. The RT interacts with an interdisciplinary network (IDN) of professionals and scientists operating as an external advisory board that validates the main steps of the project. The IDN members have been selected through an analysis of the integrative expertise needed to widen the RT institutional and operational perspective in critical aspects of public health services' management, daily veterinary professional practice, CE and leishmaniasis control programs, and One Health research and intervention planning (Table 1).

**Table 1:** Project's research team members (RT) and supervisors of the external interdisciplinary network (IDN): affiliations, background, expertise, and contributions.

Member	Affiliation	Disciplinary background	Specific expertise	Contribution to the project
RT1	Academic institute (Italy)	Vet. medicine, parasitology	Epidemiology of CE and CanL	Project planning, project coordination
RT2	Academic institute (Italy)	Agricultural economics	Agricultural and animal health economics	Project planning, economic evaluation
RT3	Academic institute (Italy)	Agricultural economics	Agricultural and animal health economics	Project planning, economic evaluation
RT4	Academic institute (Italy)	Vet. medicine, epidemiology	Epidemiology and modelling	Conceptualization of epidemiologic aspects
RT5	Vet. public health and research institute (Italy)	Vet. medicine, parasitology	Epidemiology and Diagnostics related to CE and CanL	Conceptualization of veterinary aspects
RT6	Hospital research institute (Italy)	Hum. medicine, tropical diseases	Epidemiology and clinical aspects of Human CE	Conceptualization of human CE aspects
RT7	Hospital research institute (Italy)	Hum. medicine, tropical diseases	Epidemiology and clinical aspects of HumL	Conceptualization of HumL aspects
IDN1	Regional health authority (Veneto)	Hum. medicine, management	Preventive human health	Decision-makers perspective for human public health
IDN2	Regional health authority (Veneto)	Vet. medicine, management	Veterinary public health	Decision-makers perspective for veterinary health
IDN3	Local health authority (Verona District)	Vet. medicine	Veterinary public health, animal health	Perspective of local veterinary services
IDN4	Private professional (Italy)	Vet. medicine	Professional veterinarian (pet sector)	Perspective of dog owners' and vet. private practice
IDN5	National Institute of Health (Italy)	Epidemiology, public health	CE (international expert)	Perspective from a wide experience of CE control
IDN6	National Institute of Health (Italy)	Epidemiology, public health	Leishmaniasis (international expert)	Perspective from a wide experience of leishmaniasis control
IDN7	Academic institute (UK)	Vet. medicine, vet. economics, One Health	One Health (international expert)	One Health perspective and overall revision

The EEMs explain the transmission mechanisms of CE and leishmaniasis in the region and their costs. Conceptually, the models are based on the interrelations between epidemiology and health economics [[24], [25], [26]]: the spread of pathogens in animals and humans and the distribution and intensity of symptoms cause efficiency losses that can be evaluated in economic terms by overlapping the disease epidemiology with the economic functions of the impacted entities [[27], [28], [29], [30]]. This exercise supports decision making for cost-effective health interventions.

The EEMs have been built as systems within the boundaries set by the study objectives, by identifying the relevant functional elements and the feedbacks determining the CE and Leishmaniasis outcomes and the related costs. This process resulted from the RT and IDN interaction. The first tentative models were submitted by the RT to the IDN members. Based on the comments received, revised versions were elaborated, resubmitted and then discussed between the RT and the IDN in a one-day meeting, where the final EEMs were approved.

**Fig. 1:**
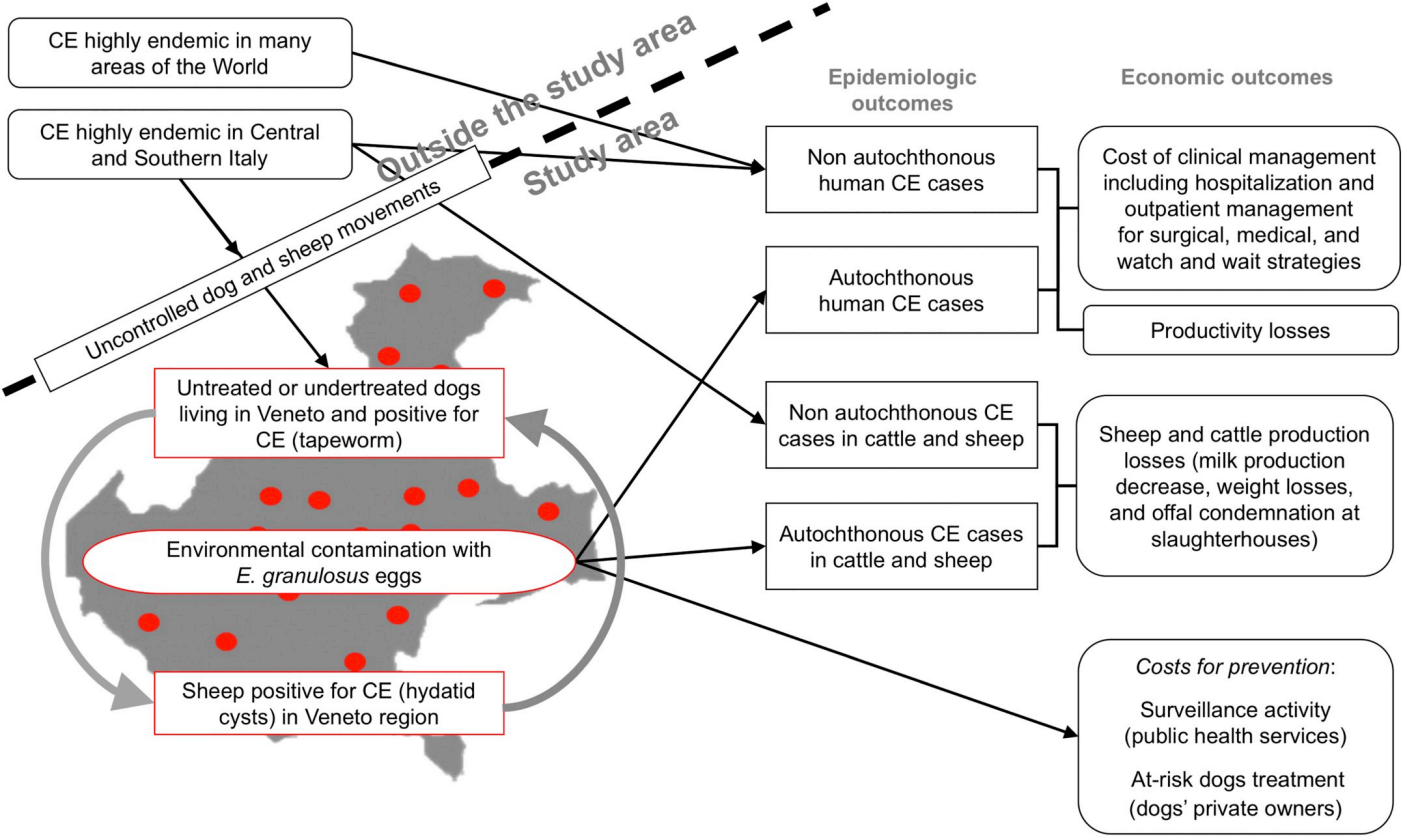
Epidemiologic-economic model flowchart of cystic echinococcosis in Veneto.

**Fig. 2:**
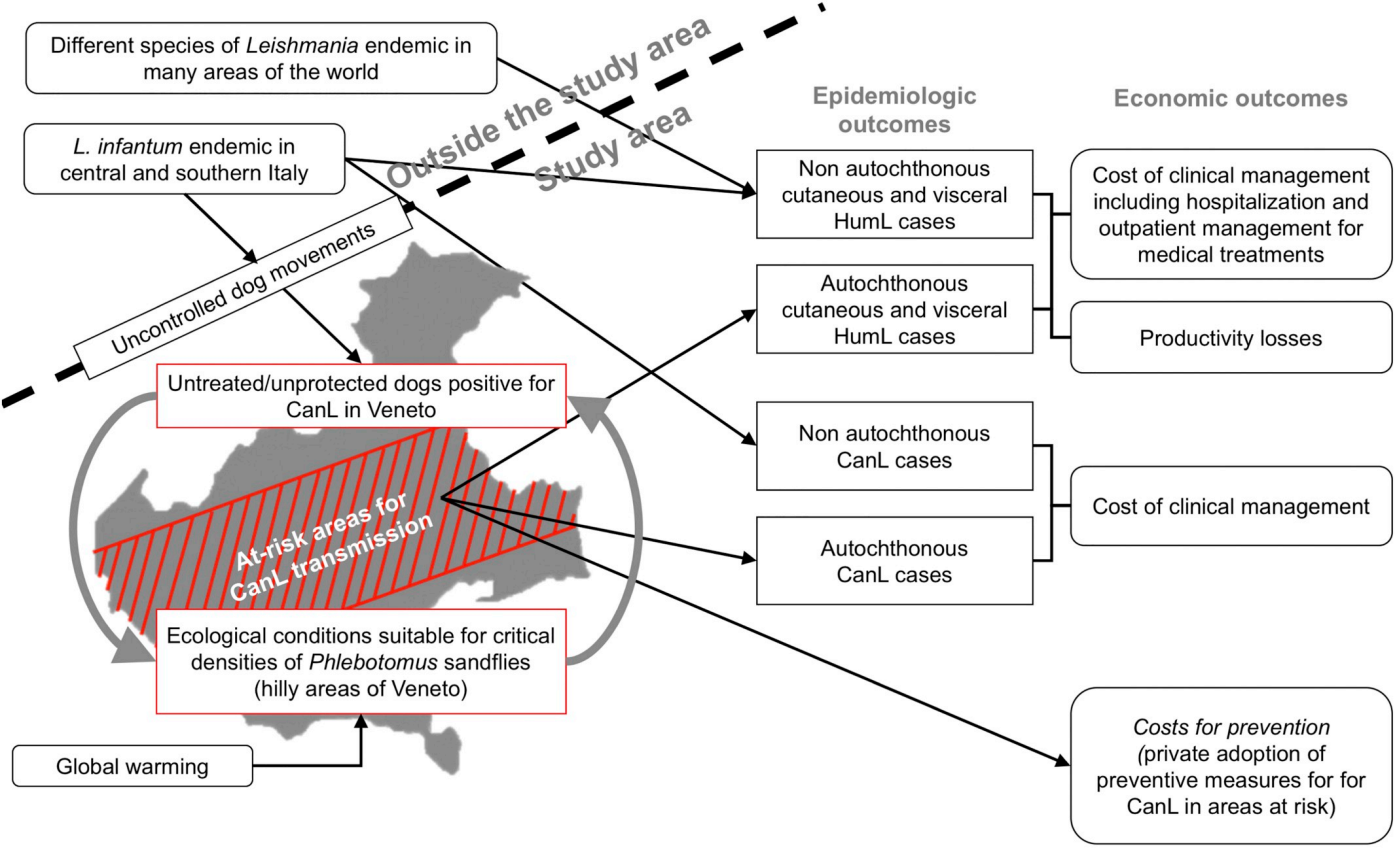
Epidemiologic-economic model flowchart of leishmaniasis in Veneto.

Two flowcharts (Fig. 1, Fig. 2) show the cause-effect links within the EEMs. This visual approach, well-grounded in epidemiology [31] and many applications of the systems theory [32,33], boosted the transdisciplinary system-building process. The EEMs allow a qualitative identification of CE and leishmaniasis costs in the region and the type of data needed to develop the following stages of the project that will be validated by the IDN through similar iterative procedures.

## 3 Cystic echinococcosis: epidemiologic-economic model and data collection strategy

In the CE flowchart (Fig. 1) the distinction between the epidemiological factors originating inside and outside the study area, represented by a map of the region, is indicated by a dashed line. The origin of the first CE animal infections in the region is unknown. Presently, around one hundred autochthonous cases of bovine CE are detected by local slaughterhouses every year [4]. Despite CE is supposed to affect mainly sheep in Veneto, no data on this species are available from slaughterhouses. Dogs positive for *E. granulosus* have been found in the last years through active surveillance [9]. Bovine CE is evenly distributed throughout the region [4], supporting the hypothesis of a moderately widespread environmental contamination of *E. granulosus* eggs (represented by red dots in the figure) that are a source of human infections. The cited studies prove the presence of an autochthonous cycle of the parasite (represented in Fig. 1 with the grey arrows connecting the boxes of positive dogs and positive sheep), hence possible local transmission to humans. Few human CE cases are treated in Veneto hospitals every year and the origin of such infections is largely unknown. The majority of patients come from other countries or Italian regions endemic for CE [10,[34], [35]].

Data collection aims at estimating the annual economic burden of CE in the region. Considering the emerging nature of the disease, hence its low prevalence in the region, the RT and the IDN agreed that data search will cover over ten years to detect temporal and spatial trends of the infection. Given the impossibility to distinguish with certainty the autochthonous from the non-autochthonous human cases, the IDN accepted the RT assumption that a CE patient resident in Veneto and born in the region or another non-endemic area will be considered an “autochthonous case”. For slaughtered cattle and sheep, animal movements can be tracked in the veterinary information system to operate this distinction.

For the evaluation of human CE costs, patients will be classified according to the three main disease management protocols: surgical, medical, and “watch and wait”. The relevant animal CE costs are related to weight losses, decreased milk production and offal condemnation in cattle and sheep, and to the treatment of dogs at risk. Table 2 shows a synthesis of the CE data collection strategy based on the relevant cost categories as settled between the RT and the IDN. Other possible costs related to the current level of the infection in the region have been considered negligible.

**Table 2:** Data collection strategy for the cost evaluation of cystic echinococcosis and leishmaniasis in the analysed region.

Type of cost	Data needed for the economic evaluation (analysed period 2001–2017)		Data sources
	Cystic echinococcosis (CE)	Leishmaniasis	
Costs for human health care;	Number of human CE cases in residents in the analysed region by age, sex, and nationality; distribution of liver CE, lung CE, and other potential localizations;	Number of human cases in residents in the analysed region by age, sex, and nationality; distribution of cutaneous and visceral leishmaniosis;	(a)
	Diagnostic-therapeutic protocols applied and distribution of patients' management according to “watch and wait”, medical, and surgical treatment and respective outcomes;	Diagnostic-therapeutic protocols applied and distribution of patients' management according to the cutaneous and visceral forms and respective outcomes;	(a) (b) (c)
	Total cost per patient for clinical management, including diagnostics, hospitalization, surgery and postoperative follow up, medical treatments and patient monitoring;	Total cost per patient for clinical management, including diagnostics, hospitalization, post-hospitalization follow-up, medical treatments and patient monitoring;	(a) (b) (c)
Productivity losses in the human population;	Patients' disability over time according to the therapeutic outcomes; gross domestic income per capita in the analysed region;	Patients' disability over time according to the therapeutic outcomes; gross domestic income per capita in the analysed region;	(a) (b) (c) (d)
Livestock production losses	Data on cattle and sheep heads, production, and production systems in the analysed region;		(c) (d) (e)
	Number of positive cases recorded in slaughterhouses for cattle and sheep (and respective livestock categories) from farms of the analysed region;		(f)
	Weight losses and milk production decrease in affected animals; livestock and livestock product prices;		(c) (g)
	Offal condemnations of positive animals at slaughterhouses; offal market prices; costs for offal disposal;		(c) (f) (g)
Costs for treatments of symptomatic dogs		Treatment protocols for symptomatic dogs; number of symptomatic dogs in the region; number of dogs treated in the region under the different protocols;	(c) (h) (i) (j)
		Unit cost of dog treatment for the different treatment protocols;	(h) (i) (k)
Costs for preventive treatment of exposed dogs	Definition of “at-risk” dog and standard preventive treatment protocol for at-risk dogs; number of at-risk dogs in the region; number of dogs subjected to preventive treatments;	Definition of a standard preventive treatment protocol; total number of dogs in the areas where the vector is present; number of dogs subjected to preventive treatments;	(c) (h) (i) (j)
	Annual unit cost for dog treatment;	Annual unit cost for dog preventive treatment;	(h) (i) (k)
Costs for surveillance of susceptible livestock species;	Notifications from slaughterhouses of infected animals coming from the analysed region: number and unit costs;		(i) (l)
	Investigations on farm outbreaks in the region by the public veterinary services: number and unit costs;		(i) (l)
	Costs related to registrations, periodical reporting and maintenance of the information system;		(i) (l)
(a) Data from the hospital discharge records (HDRs) (b) Administrative and financial records of a reference hospital in the analysed region; (c) Information and data from the scientific literature; (d) Statistics on population, economy, and agriculture in the analysed region; (e) Data from the national veterinary information system; (f) Survey on Italian slaughterhouses receiving animals from the analysed region; (g) Agricultural price bulletins;		(h) Interviews with private practice veterinarians managing animal health in farms and veterinary clinics for pets; (i) Interviews with veterinarians of the public health services (j) Data from the national canine registry; (k) Price lists of veterinary medicines; (l) Administrative and financial records from the veterinary public health services.	

## 4 Leishmaniasis: epidemiologic-economic model and data collection strategy

The leishmaniasis EEM also distinguishes the epidemiological factors originating within and outside the study area (Fig. 2).

The parasite was introduced from outside the region, but an autochthonous circulation of L. *infantum* (represented by the grey arrows connecting the boxes of positive dogs and sandfly high density in Fig. 2) has been demonstrated for many local canine populations [8,36].

Ecological conditions suitable for the development of the vector population can be found in the hilly parts of Veneto [37], where CanL endemicity is then favoured. In these zones, the territory of the municipalities with demonstrated parasite circulation in dogs (roughly represented by red diagonal stripes in Fig. 2) has been delimited as the area “at risk”. The rest of the region seems unapt for the development of critical sandfly densities. However, a progressive future expansion of the area at risk is possible because of global warming.

Few human cases of cutaneous and visceral HumL are reported each year from Veneto hospitals, but their origin is mostly uncertain [11]. The RT and IDN then agreed that one patient residing in a municipality within the area at risk and born in Veneto or another non-endemic region will be considered an “autochthonous” HumL case. A distinction between cutaneous and visceral forms will be applied for the HumL cost evaluation. Costs for CanL concern the treatment of symptomatic dogs and the prevention of CanL in the areas at risk. The RT and IDN decisions about leishmaniasis data collection strategy and main cost categories to be considered are summarised in Table 2.

## 5 Final considerations

The holistic approach of One Health supports the idea that economic evaluations of health interventions should consider the complexity of the human, animal and environmental context to a broad extent, hence the need for a methodological shift toward inter/trans-disciplinarity and systems thinking [38]. Fulfilling its first stage, this research shows the feasibility of such a shift, even with small-scale projects and limited financial resources. The elaboration of the two EEMs tested the possibility of setting innovative procedures between researchers and institutions from different fields. Three aspects of this exercise should be underlined:

1. the integration of expertise resulting in an effective interdisciplinary work to identify the main cost categories and plan data collection for a comprehensive economic evaluation;
2. the cooperation within and between the RT and the IDN leading to the validation of the EEMs in a wider perspective than that achievable by a mono-disciplinary institution or group;
3. the optimization of available resources, i.e. individual skills, professional roles and institutions already working on different aspects of the same problems, but usually not coordinated toward specific goals, especially when dealing with neglected zoonoses like CE and leishmaniasis.

The definitions of “autochthonous” human cases for the two diseases, based on information from veterinary surveillance (e.g. the delimitation of areas at-risk for HumL based on CanL endemicity) are examples of fruitful interdisciplinary expertise integration and institutional cooperation driven by a One Health approach. The first results of this project indicate that One Health methods, although still pioneering or unusual in many scientific and professional contexts, can be implemented with limited resources and already available capacities.

## Author contributions

MC, MA, and RC conceived the project and drafted the manuscript. AA, GC, MD, FG, and FT contributed to the development of the epidemiologic-economic models and manuscript revision and editing.

## Declaration of Competing Interest

The authors declare that the research was conducted in the absence of any commercial or financial relationships that could be construed as a potential conflict of interest.
